# HOW TO PREPARE PHYSICIAN ASSISTANTS FOR THE CARE OF OLDER ADULTS? PARTNER WITH GERONTOLOGY

**DOI:** 10.1093/geroni/igz038.1095

**Published:** 2019-11-08

**Authors:** Jacqueline Eaton, Trenton Honda

**Affiliations:** University of Utah, Salt Lake City, Utah, United States

## Abstract

Approximately 80% of older adults have chronic illness which requires complex care. Primary care providers require special training to improve the care they provide older adults. As primary care shortages increase, and the older adult population swells, physician assistants (PA) will increasingly be relied upon to provide care and advocacy for older adults. The purpose of this presentation is to describe the development of a dual degree program that facilitates enhanced gerontological training for students pursuing a Masters of PA Studies degree. The Gerontology Interdisciplinary Program and the PA Program at the University of Utah collaborated to assess program objectives, competencies, and coursework, while identifying student interest in this dual MS venture. Students were interested in increasing their skills to meet the growing need for geriatric care while also saving time and money. In addition, it was important that the combined program of study did not overburden students and accommodated participants off-site. Faculty and administrative buy-in was sought from within departments and colleges. Revisions to the proposed program of study included altering course offerings, changing program start dates, and removing course overlap. A 30-month dual MS was developed that incorporates 87 PA Program credits, 25 Gerontology Credits, and 6 credits shared through practicum, geriatric content, and evidence based practice. Students who graduate will complete a Gerontology focused Masters Project that combines their work from the two programs. This dual MS program prepares students to be competitive in the job market, while also targeting an area of need in primary care.

